# Sorting Five Human Tumor Types Reveals Specific Biomarkers and Background Classification Genes

**DOI:** 10.1038/s41598-018-26310-x

**Published:** 2018-05-25

**Authors:** Kimberly E. Roche, Marvin Weinstein, Leland J. Dunwoodie, William L. Poehlman, Frank A. Feltus

**Affiliations:** 1 Clemson University, Department of Genetics & Biochemistry, Clemson, 29634 SC USA; 2 Quantum Insights Inc., Menlo Park, 94025 California USA

## Abstract

We applied two state-of-the-art, knowledge independent data-mining methods – Dynamic Quantum Clustering (DQC) and t-Distributed Stochastic Neighbor Embedding (t-SNE) – to data from The Cancer Genome Atlas (TCGA). We showed that the RNA expression patterns for a mixture of 2,016 samples from five tumor types can sort the tumors into groups enriched for relevant annotations including tumor type, gender, tumor stage, and ethnicity. DQC feature selection analysis discovered 48 core biomarker transcripts that clustered tumors by tumor type. When these transcripts were removed, the geometry of tumor relationships changed, but it was still possible to classify the tumors using the RNA expression profiles of the remaining transcripts. We continued to remove the top biomarkers for several iterations and performed cluster analysis. Even though the most informative transcripts were removed from the cluster analysis, the sorting ability of remaining transcripts remained strong after each iteration. Further, in some iterations we detected a repeating pattern of biological function that wasn’t detectable with the core biomarker transcripts present. This suggests the existence of a “background classification” potential in which the pattern of gene expression after continued removal of “biomarker” transcripts could still classify tumors in agreement with the tumor type.

## Introduction

Dozens of public genomic data repositories relevant to human biology have emerged to support biomedical science. These repositories include The Cancer Genome Atlas (TCGA^1^), the Genotype-Tissue Expression (GTEx) project^2,3^, and the Database of Genotypes and Phenotypes (dbGaP^4^). These databases are deep and diverse, containing data ranging from DNA sequence to epigenetic state to dynamic gene expression output. TCGA, for example, contains multiple measurements for over 14,000 tumors of multiple types. GTEx contains the gene expression patterns and matching DNA sequences for over 11,000 human tissue samples. dbGaP contains over 2.4 million molecular assays relevant to human disease and phenotypes. Clearly, there is massive opportunity to mine these and future databases for biological insight.

A powerful quantitative measurement of genome information flow within a biological sample is the steady state RNA-based gene expression profile; this profile is captured in a gene expression vector representing the number of RNA molecules produced by tens of thousands of genes in the specimen. A researcher can aggregate profiles from multiple samples retrieved under varied biological contexts into *m* × *n* gene expression matrices (GEMs) where rows (*m*) are gene or RNA transcript identifiers and columns (*n*) are samples. One can think of a GEM as a compendium of molecular snapshots from different points of anatomy, developmental stage, and environment. While a liver gene vector, for example, has the same genes as a neighboring pancreas gene vector, their gene expression intensities vary in a manner reflective of their underlying biology.

GEMs can be normalized to reduce technical variation between biological samples^3,5,6^. Correlation analysis can be performed to identify genes whose expression patterns are synchronized across samples using software such as WGCNA^7^ and KINC^8^. Similar gene expression patterns are predicted to have a common biological purpose. However, the brute force interrogation of all samples in a GEM may not be the best approach to detect context-specific gene interactions; overrepresentation of one biological condition might drown out rare gene interactions. One approach to addressing variation between samples involves sorting the gene expression vectors into sample clusters of similar contexts. Thus, liver expression profiles might produce one sample cluster, as will pancreas profiles, etc. Correlation analysis can be performed in sorted sample clusters. Sample sorting is becoming increasingly relevant given the rapid growth of samples in databases that are being interrogated for biological function. If a sample sorting technique can robustly sort mislabeled or outlier samples in to alternate groups, then noise should be reduced for each group possibly making it easier to identify meaningful biomarkers and pathways.

Two approaches exist for clustering GEMs into sample groups of similar global gene expression patterns: knowledge-dependent and knowledge-independent methods. Both can increase the probability of detecting gene expression patterns relevant to the sorted biological contexts. Knowledge-dependent methods sort the GEM into sub-GEMs based upon annotations associated with the samples. Thus, one could prepare a mixed knowledge-independent condition GEM from a data repository like TCGA and then sort the GEM into tumor types prior to analysis. This approach, while logical – the metadata associated with TCGA is well curated – weakens if the sample label is assigned incorrectly or is representative of multiple subgroups. This can happen, for example, when a tumor type is incorrectly annotated or when tumors are related by molecular architecture as opposed to tissue of origin^9^.

A less-biased, knowledge-independent approach uses clustering methods to sort datasets into groups based only upon their global expression pattern. For example, k-means clustering of global gene expression profiles sorts samples into a pre-defined number of groups and has been shown to improve gene-gene interaction detection^10–12^. Sample metadata is assigned after sorting to provide conditional context to the clusters. One sample cluster might be enriched for a specific biological context label, such as a tumor type, and any genetic relationships from that cluster can be associated with that label. A drawback to k-means clustering is that the number of clusters should be known beforehand, resulting in bias in the sample grouping if an inappropriate number of distinct clusters is chosen.

There are emerging knowledge-independent clustering methods that can be applied to GEMs and that do not introduce as much bias. One method, t-distributed stochastic neighbor embedding (t-SNE^13,14^) – like most knowledge independent sample clustering approaches – relies upon strongly reducing the dimensionality of the gene expression space prior to sample comparison. Two common algorithms used to perform this task are principal component analysis (PCA^15^) and singular value decomposition (SVD^15^). Typically, this machine learning technique projects high dimensional data into two or three dimensions. It should be noted that t-SNE has been applied to GTEx^16^ and TCGA^17^ datasets, where the TCGA study used t-SNE as part of an integrated omics sorting workflow called MEREDITH^17^.

Dynamic Quantum Clustering (DQC^18^), unlike other clustering approaches, does not need to use strong dimensionality reduction to analyze high-dimensional data; however, it is common in a DQC analysis to use modest SVD-based dimensionality reduction to speed up the analysis. DQC begins by replacing each column of a GEM (that can be thought of as a vector in an *n*-dimensional Euclidean space) by an analytic function in *m*-variables; specifically, each column of the GEM is replaced by a Gaussian function centered on the *m*-dimensional location specified by the corresponding gene expression vector. The sum of these functions is then used to create a *potential function*, *V*, that is a proxy for the density of the data in feature space^19^. By construction, the local minima and saddle points of this *potential function* represent regions of higher local density of the data. This potential function is then used to create a Hamiltonian operator as Equation (1):

1 $$H=-\,\frac{1}{2m}{\nabla }^{2}+V$$

Each individual Gaussian, *ψ*(*x*), is then evolved according to the corresponding Heisenberg equation of motion^20^ in Equation (2):

2 $$\psi (x,\,\delta t)={e}^{-i\delta tH}\,\psi (x)$$

The center of this time-dependent Gaussian will move a short distance, implementing a modified, operator form of gradient descent that moves the original center towards the nearest local minimum of *V*. Due to the non-local effect of quantum evolution, there are important differences that allow DQC to avoid the difficulties associated with simple gradient descent for many points in high dimension. In particular, choosing a low value for the mass parameter, *m*, exploits quantum tunneling to avoid getting trapped in small fluctuations, avoiding many of the issues related to working in high dimension.

Another major difference between a DQC analysis in *m*-dimensions and other data-mining methods is that the entire analysis is encoded as an *m*-dimensional animation. This visual presentation of the computation provides a detailed record of each computational stage, showing how clusters and other structures form. A benefit of the DQC approach is that it avoids introducing bias; there is no need to invent hypotheses to test, assume the number or type of clusters that exist, or invoke prior sample knowledge. Selected frames from DQC animations are shown in this report and the full animations are available for viewing in Supplementary Videos 1 and 2.

This study analyzed a mixed tumor type GEM from TCGA containing the RNAseq expression profiles of 2,016 tumors from five tumor types: lower grade glioma (LGG), glioblastoma multiforme (GBM), ovarian serous cystadenocarcinoma (OV), urothelial bladder cancer (BLCA), and papillary thyroid carcinoma (THCA). We discuss the clustering and biomarker discovery potential using t-SNE and DQC approaches. In addition, we examine the effect of salient biomarker removal and the ability of both techniques to continue to classify the tumors into meaningful groups in the absence transcripts with high classification potential. When applied to deeply sequenced tumors, our approach can be used to detect biomarker combinations that sort tumor types without prior knowledge of where the tumor was initiated.

## Results

### Tumor Separation via DQC and t-SNE Analyses

**Figure 1:**
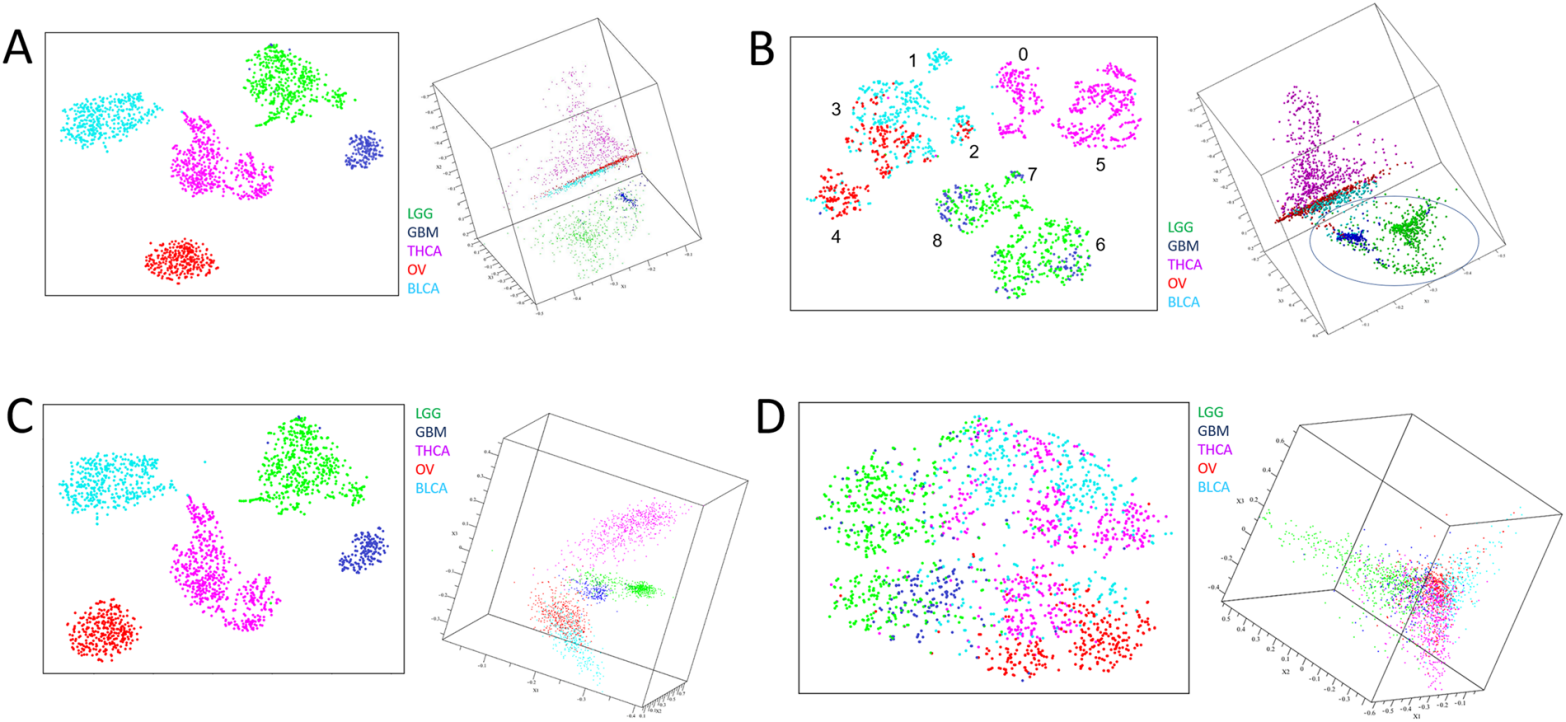
Tumor Classification Potential Revealed by t-Distributed Stochastic Neighbor Embedding (t-SNE) and Dynamic Quantum Clustering (DQC). Each point represents a tumor sample. (**A**) The full 2,016-tumor gene expression matrix (73,599-transcripts) embedded using t-SNE (left) or evolved by DQC (right); (**B**) The full 2,016-tumor gene expression matrix (48 “core transcripts”) embedded using t-SNE (left) or evolved by DQC (right). Numbers denote consensus clusters identified by HDBSCAN. We circled the brain tumor “arch” – discussed in the text – that is only seen in the DQC analysis; (**C**) The full 2,016-tumor gene expression matrix (all transcripts minus 48 “core transcripts”) embedded using t-SNE (left) or evolved by DQC (right); (**D**) The full 2,016-tumor gene expression matrix (48 random transcripts) embedded using t-SNE (left) or evolved by DQC (right). Full DQC animation of 48 “core transcripts” can be seen in Supplemental Video 2.

We first examined the clustering potential of DQC^18^ and compared it to an approach that performs strong dimensional reduction, t-SNE^13^. To do this, we constructed a mixed tumor GEM consisting of 2,016 samples from TCGA^1^. Specifically, we mixed tumor expression profiles from five TCGA labeled sample groups: bladder cancer (BLCA; n = 427), glioblastoma (GBM; n = 174), lower grade glioma (LGG; n = 534), ovarian carcinoma (OV; n = 309), and thyroid cancer (THCA; n = 572). It should be noted that some of the groups contained low numbers of non-tumor samples with the same label as tumor type (BLCA = 19/427; GBM = 5/174; LGG = 0/534; OV = 0/309; THCA = 59/572). This GEM was used as the input for both the DQC and t-SNE analyses (Fig. 1) discussed in the following sections.

We began by using DQC to produce animations showing the “quantum evolution” of the 2,016 tumor samples for the SVD-decomposition of the GEM containing all 73,599 transcripts (Fig. 1A). This initial analysis was done in both 50 and 60 SVD-dimensions. These values were chosen by the requirement that restricting to the first 50 or 60 SVD eigenvalues would approximate the original mixed GEM to better than the 1.5% level (as measured by the change in matrix norm defined as the square root of the sum of the squares of every entry in the matrix). Since the overall pattern of data-separation is evident in all dimensions after this DQC evolution, we only show plots for the first twelve SVD-dimensions (frames of dimensions 1–3 in Fig. 1). This DQC analysis produced clusters with structure in which BLCA, OV, and THCA samples formed extended flat shapes populated by many distinct sub-clusters. Note, however, that the overall shape of the region where these clusters are located only showed a slight change during DQC evolution. In contrast, the GBM and LGG samples showed obvious change with DQC evolution, first forming complex filamentary structures that then separate into two somewhat distorted clusters.

After the initial DQC-evolution, we used DQC based feature selection (see Materials and Methods for details) to discover 48 “core transcripts” (Supplemental Table 1) that produced a very similar view of the data. We then subjected a subset GEM containing just the columns corresponding to these 48 transcripts to DQC evolution (Fig. 1B) in the full 48 dimensions. Because these 48 “core transcripts” do a very good job of representing the important structure in the data, the DQC results are very similar to the DQC evolution of the full GEM. As before, BLCA, OV, and THCA samples formed planes in three dimensions occupied by many distinct clusters. However, evolution of the GEM restricted to the “core transcripts” revealed that the GBM and LGG samples formed a “brain arch” with GBM samples distributed at one extreme and LGG along the rest of the structure (circled in Fig. 1B). This strong separation of GBM from LGG is evidence that – at least for glial cell tumors – the 48 “core transcripts” are providing information about tumor type and not just tissue of origin. It should be noted that only the DQC analysis revealed a strong separation of GBM and LGG tumors; the tSNE-analysis divided these tumors into several clusters, but none of these sub-clusters were highly enriched for a specific tumor type.

The geometry visible in the previous plot changes markedly when the TCGA matrix is analyzed with the 48 core transcripts removed (Fig. 1C). In this case the shape of the data is entirely different, in that it exhibits four clearly separated lobes. Still, DQC evolution of this dataset showed that all five tumor types could be easily separated from one another. This surprising result led to further study described later in this report. Finally, to test the efficacy of DQC-based feature selection, we showed that repeated selection of 48 random transcripts did not separate the tumors into tissue of origin by t-SNE or DQC (representative result in Fig. 1D).

Sample clustering of the full TCGA GEM was repeated using the t-SNE-HDBSCAN pipeline (Fig. 1A–D). Like DQC, t-SNE segregated the tumors into five groups using all 73,599 transcripts (Fig. 1A), the 48 core transcripts (Fig. 1B), or all transcripts minus the 48 core transcripts (Fig. 1C). Forty-eight random transcripts did not cluster the tumors via t-SNE (Fig. 1D; 1/20 runs shown with similar result). The 48-core transcript subset segregated the five tumor types into nine clusters in two dimensions as identified using the HDBSCAN/cluster ensembles consensus clustering approach (Fig. 1B). Consensus clusters are labeled as numbers in Fig. 1B and discussed below. In contrast with DQC, the 48 core transcripts failed to produce t-SNE embeddings that cleanly separated the five tumor types, failed to cleanly separate GBM from LGG, and failed to reveal the “brain arch” (Fig. 1B) although in general t-SNE embedding produced clusters that segregated more distinctly than DQC.

### The “Brain Arch” Tumor Substructure

**Figure 2:**
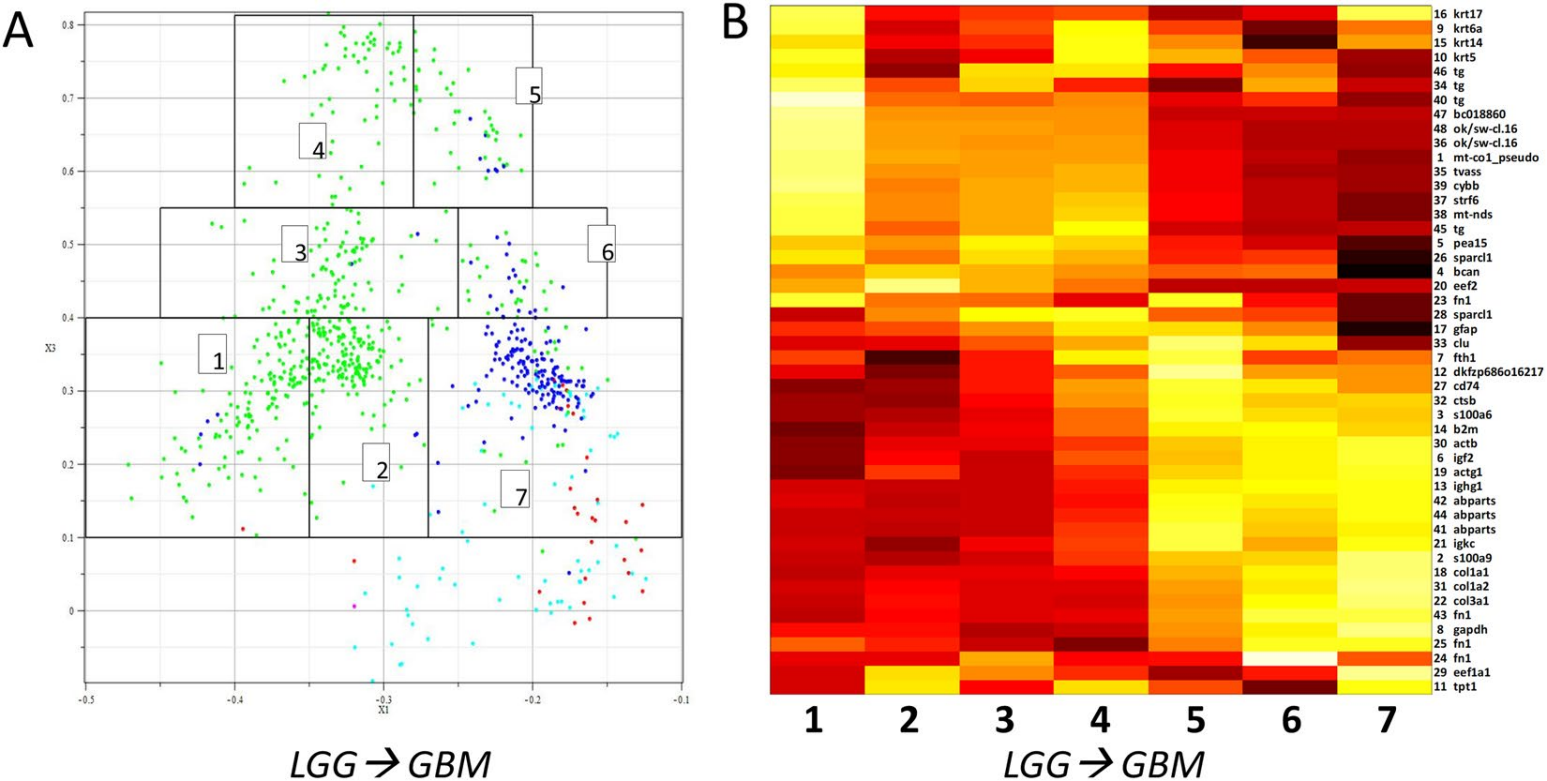
LGG and GBM Samples Create Complex “Brain Arch” Structure in DQC Evolutions. (**A**) The 48 “core transcript” subset of LGG (green) and GBM (blue) samples produces an arch in DQC. Segmentation of samples within the arch was performed by k-means clustering at frame 56 of the DQC evolution of the 48 transcripts. Each arch group was labeled with a number; (**B**) Average gene expression heatmap of all 48 transcripts along the arch for each position numbered to the left. Yellow is high expression and red is low expression. Locus names (Supplemental Table 1) are shown on the far right.

DQC analysis revealed an interesting substructure between LGG and GBM brain tumors. To examine this structure in more detail, we dissected the arch samples into seven groups by k-means clustering (Fig. 2A). Groups 1 through 5 are primarily LGG tumors while groups 6 and 7 are GBM. Interestingly, visualizing the expression levels across the “brain arch” shows a trend of epithelial, thyroid, and other genes turned on at the left-hand leg of the arch (LGG-enriched group 1) which tend to be off at the right-hand leg (GBM-enriched group 7). There also appears to be immune response, extracellular matrix, and differentiation genes turned on at the bottom of the right leg (GBM-enriched group 7 of the arch and off at the bottom of the left leg (LGG group 1)).

### Annotating Tumor Consensus Clusters

**Figure 3:**
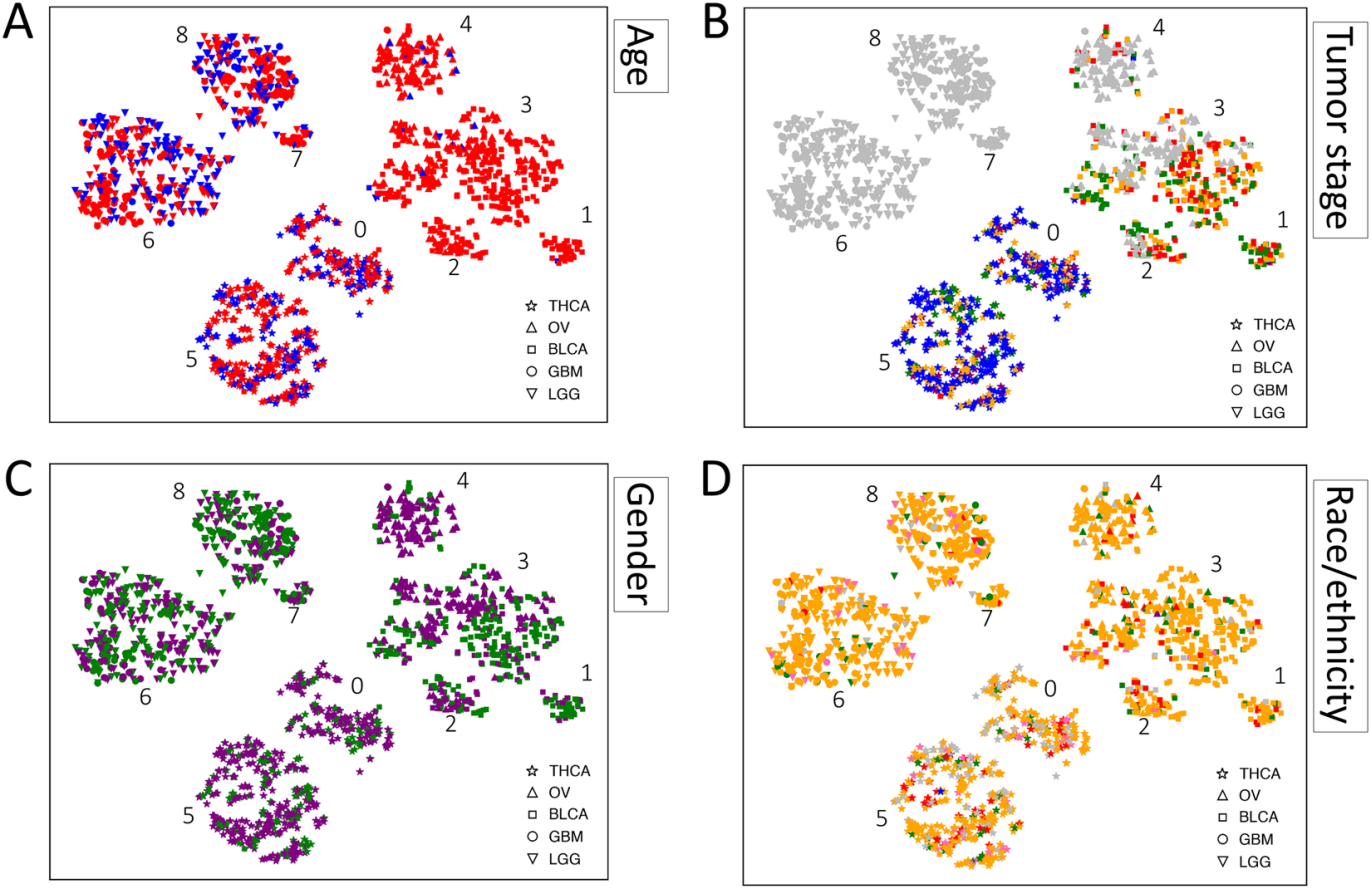
t-SNE Embeddings of 48 Transcripts Color-coded for Patient Attributes. (**A**) Age: red = “age at or above 40”, blue = “age below 40”; (**B**) Race/ethnicity: blue = “American Indian or Alaska native”, red = “Asian”, green = “Black or African American”, purple = “native Hawaiian or Pacific islander”, pink = “Hispanic or Latino”, yellow = “white”; (**C**) Gender: green = “male”, purple = “female”; (**D**) Tumor stage: blue = “stage I”, green = “stage II”, yellow = “stage III”, red = “stage IV”, purple = “stage IV-A/B/C”. Gray in all cases indicates missing data. Note the embedding of the samples used here is different than Fig. 1B but the cluster identities are identical.

**Figure 4:**
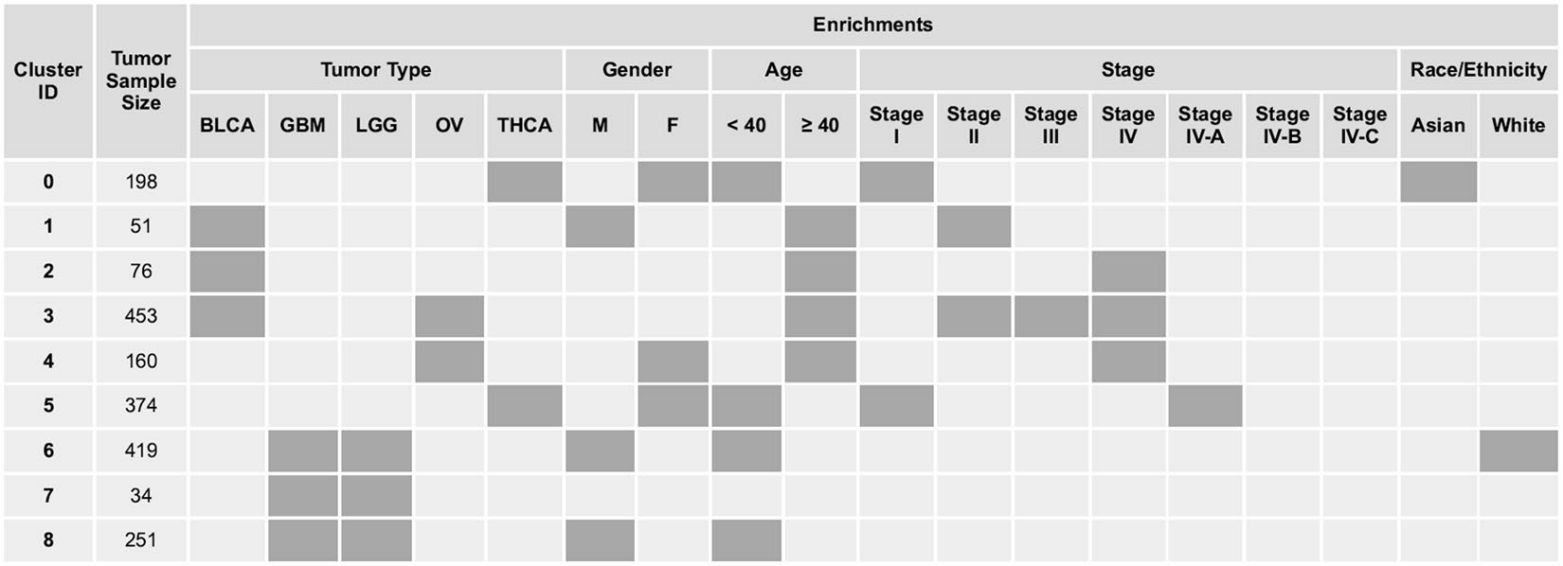
t-SNE Embedding-derived Patient Attribute Enrichment. Consensus cluster identification and attribute enrichment results from 1000 t-SNE embeddings of the 48 transcripts. Gray boxes indicate significant enrichment (p < 0.001).

Tumors that cluster by gene expression pattern would be expected to show enrichment for tumor type label and other attributes. Enrichment analysis was performed on the consensus clusters labeled in Fig. 1B (p < 0.001). TCGA patient attributes exist for age, race, ethnicity, gender, and tumor stage were available for the majority of the 2,016 tumor samples. t-SNE embeddings of the 48 core transcripts with patient attribute values labeled by color are shown in Fig. 3 and listed in Fig. 4.

All clusters were statistically enriched (p < 0.001) for at least one tumor type label. Clusters 6, 7, and 8 were enriched for both GBM and LGG tumor type labels. Thyroid tumor clusters were enriched for the female gender. One of the bladder tumor clusters and two of the mixed brain tumor clusters were enriched for the male gender. The bladder and ovarian tumor clusters were uniformly enriched for age above 40 years, while several of the thyroid and brain tumor clusters were enriched for age below 40. The age enrichment corresponded roughly to stage enrichment: both thyroid clusters were enriched for tumors in their earliest stage while the mixed BLCA-OV clusters 2, 3, and 4 (where data was available) were enriched for stage IV tumors. Little enrichment was observed based on race or ethnicity although thyroid-enriched cluster 0 was also enriched for “Asian” as a race. The mixed GBM-LGG tumor cluster 6 was enriched for the annotation term “white”. It is worth noting that we did not attempt to correct for bias that may exist within the TCGA database itself with regard to sample collection.

### Tumor Classification Potential by DQC Based Transcript Selection and Removal

As can be seen in Fig. 1C, the initial reduced GEM (*i.e*. the one obtained from the original GEM by removing the 48 core transcripts) can still be used to successfully segregate the tumors into five groups via either t-SNE or DQC. Moreover, the DQC analysis once again successfully separated the GBM tumors from the LGG tumors. So, we see that – at least for the case of glial cell tumors – information about tumor type and not just tissue of origin is being encoded in the reduced GEM. Still, brain tumors aside, the observed DQC and t-SNE substructure both distinguished the other tumors. Why would classification occur in the absence of the core biomarkers? It is well known that hundreds to thousands of genes might be differentially expressed between tumor types. In the case of GBM and LGG, it has previously been reported that 2275 genes are differentially expressed between these tumor types^21^. Therefore, one would expect several thousand transcripts to have a combined sorting ability as the gene set without core biomarkers is still likely embedded with differentially expressed genes. However, we were curious about the effect of deeper biomarker removal and the effect on sample sorting. Thus, we performed successive rounds of DQC-based feature (*i.e*. important transcripts) selection and removal.

**Figure 5:**
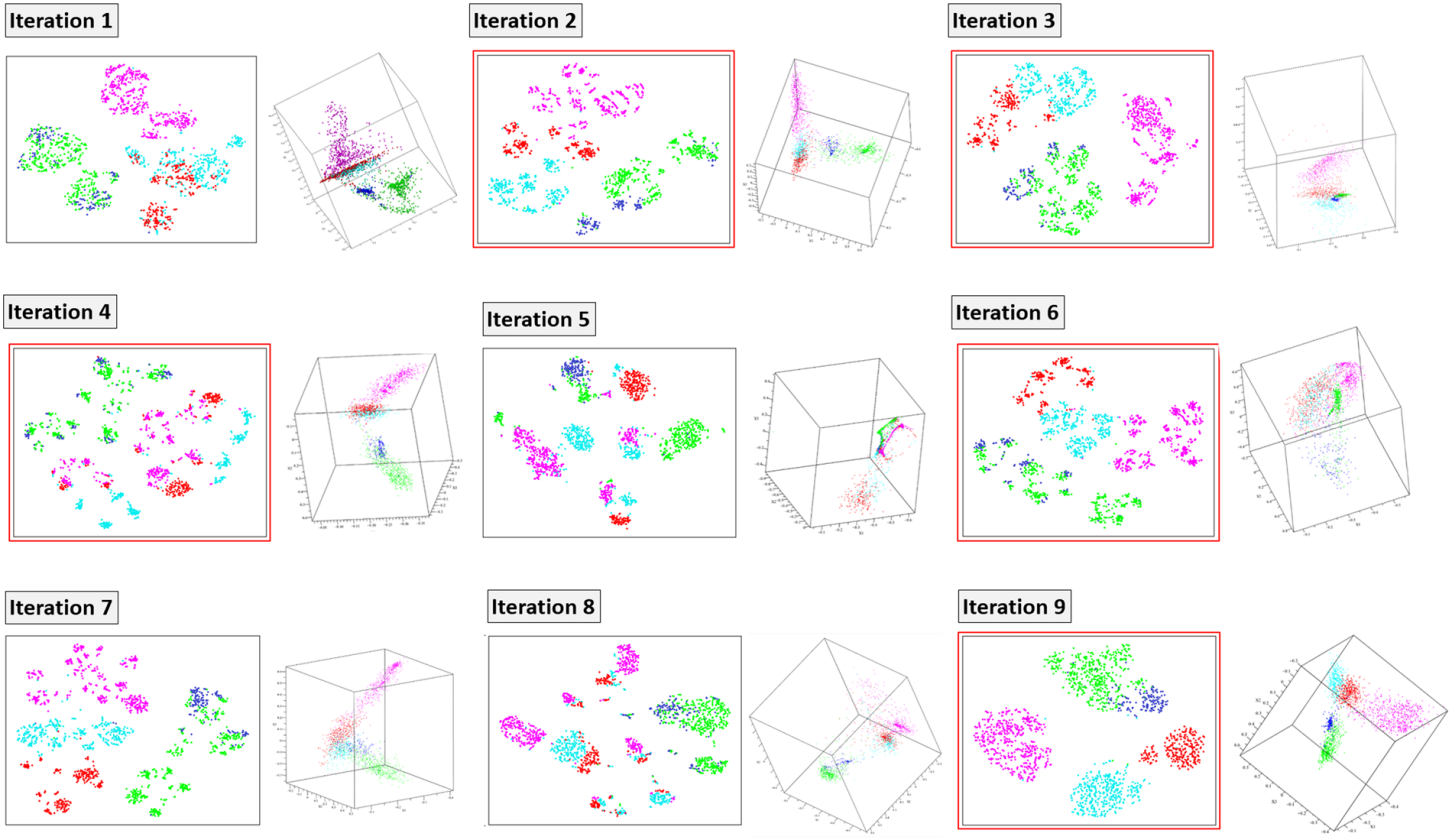
Layer Extraction by DQC Based Feature Selection. Eighteen iterations of DQC based feature selection identified transcripts in windows of decreasing information content. The first nine iterations are shown with a representative t-SNE embedding on the left and a DQC snapshot in the first three dimensions on the right for each iteration. Iteration 1 is the same data as shown in Fig. 1B. The number of transcripts identified with SVD-entropy from iteration 1 to 18: 48, 57, 55, 22, 22, 73, 54, 23, 146, 87, 41, 34, 413, 279, 209, 119, 80, 2102. Iterations in red show repeating patterns in Fig. 7.

**Figure 6:**
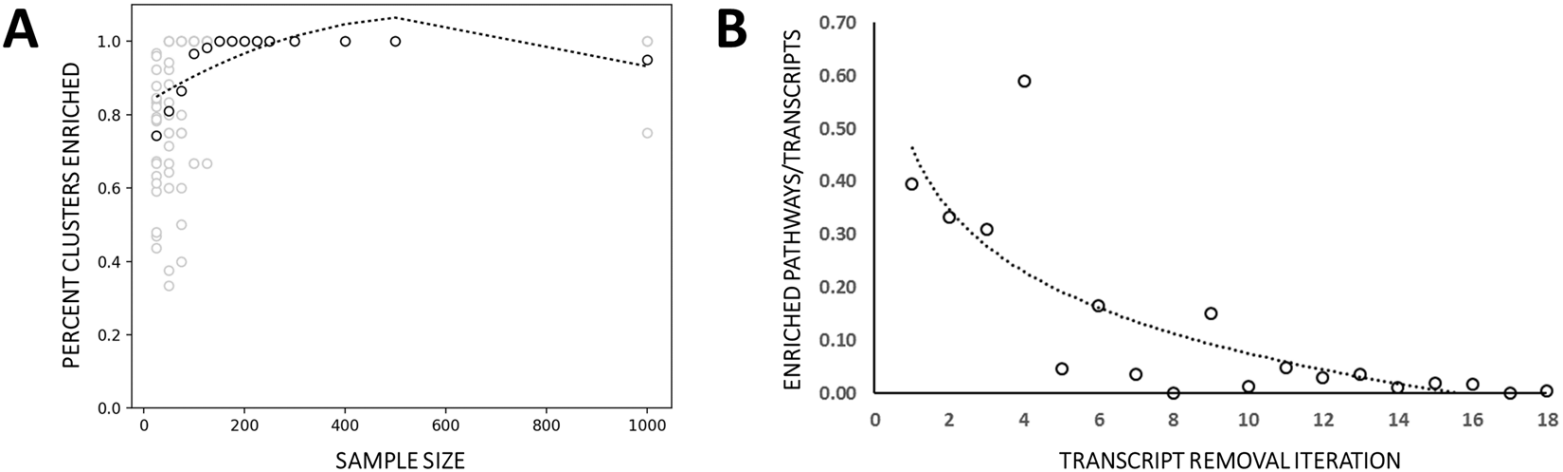
Cluster Coherence Increases with Sample Size and Functional Enrichment Decreases with Decreasing DQC Feature Selection Threshold. (**A**) Percent of clusters enriched for at least one tumor type increases with sample size; (**B**) The number of Reactome pathways at each level of feature selection corrected for transcript count.

Since this part of the analysis was only focused on how systematic removal of features would affect the ability to separate tumors, we decided to expedite the analysis by limiting DQC-based feature selection to the first 12 SVD dimensions to identify the next layer of important transcripts. For these 12 eigenvectors, we plotted the sorted absolute values of their components and set a threshold based upon breaks in the plot. These thresholds steadily decreased as we repeated the process of important transcript selection and removal. At each iteration, we checked that the new set of important transcripts alone could still separate the glial cell tumors by diagnosis (Fig. 5) and not just tissue of origin. Furthermore, we tested the gene set for the significant enrichment of biological annotation terms (Fig. 6; Supplemental Table 2), removed the important transcripts, and then repeated the transcript selection process. We repeated this procedure a total of 18 times, thus identifying multiple layers of important transcripts corresponding to relaxed threshold magnitudes for the SVD-eigenvectors.

Remaining transcripts after iterative removal of important transcripts were studied by both DQC and t-SNE (iterations 1–9 shown in Fig. 5). All iterations reveal varying capacity of the remaining transcripts to segregate the five tumor types. The number of transcripts in each interval also varied greatly. The number of transcripts identified at each level - from iteration 1 to 18- are 48 (core transcripts from the full GEM described above), 57, 55, 22, 22, 73, 55, 23, 146, 87, 41, 34, 413, 280, 209, 119, 80, 2101. The iterations with least number of transcripts, obtained in iterations 4 and 5, identified 22 RNA transcripts each; the largest, at 2101 transcripts, had significant tumor “classification potential” despite the population of transcripts being the least sensitive as measured by difference of absolute value of the components of the SVD-eigenvectors. We performed DQC evolutions for iterations 1–9 but due to size constraints, a single example DQC evolution for iteration 9 can be found in Supplemental Video 1.

**Figure 7:**
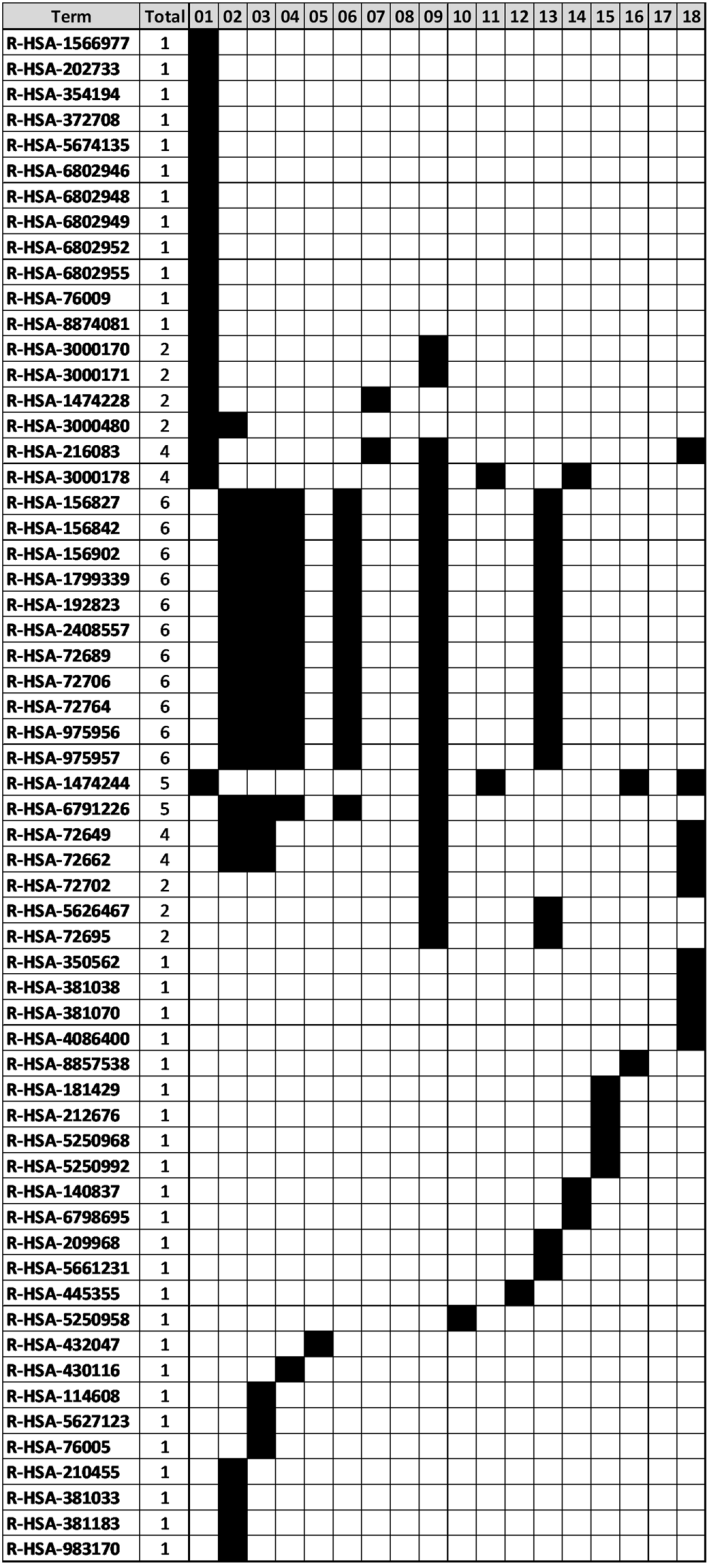
Biological Enrichment at Each Layer of SVD-Entropy Threshold Relaxation. Specific Reactome pathway enrichment (FDR < 0.01) at each level of feature selection (01–18) is shown as a black box. The total number of shared functions across all magnifications is shown.

In this analysis, we noticed a trend where, for both DQC and t-SNE analyses, layers with larger transcript population size generally exhibit greater capacity for tumor segregation (Fig. 6A). In contrast, performing biochemical pathway enrichment analysis on the transcripts at each level, we see a decrease in biological function (*i.e*. enriched Reactome pathways; Fig. 6B) at each level. While the first core transcript iteration contains a complex set of tumor type and tissue relevant pathways, iterations 2, 3, 4, 6, 9, and 13 repeat identical pathways (Fig. 7).

### “Background” Tumor Classification Potential

**Figure 8:**
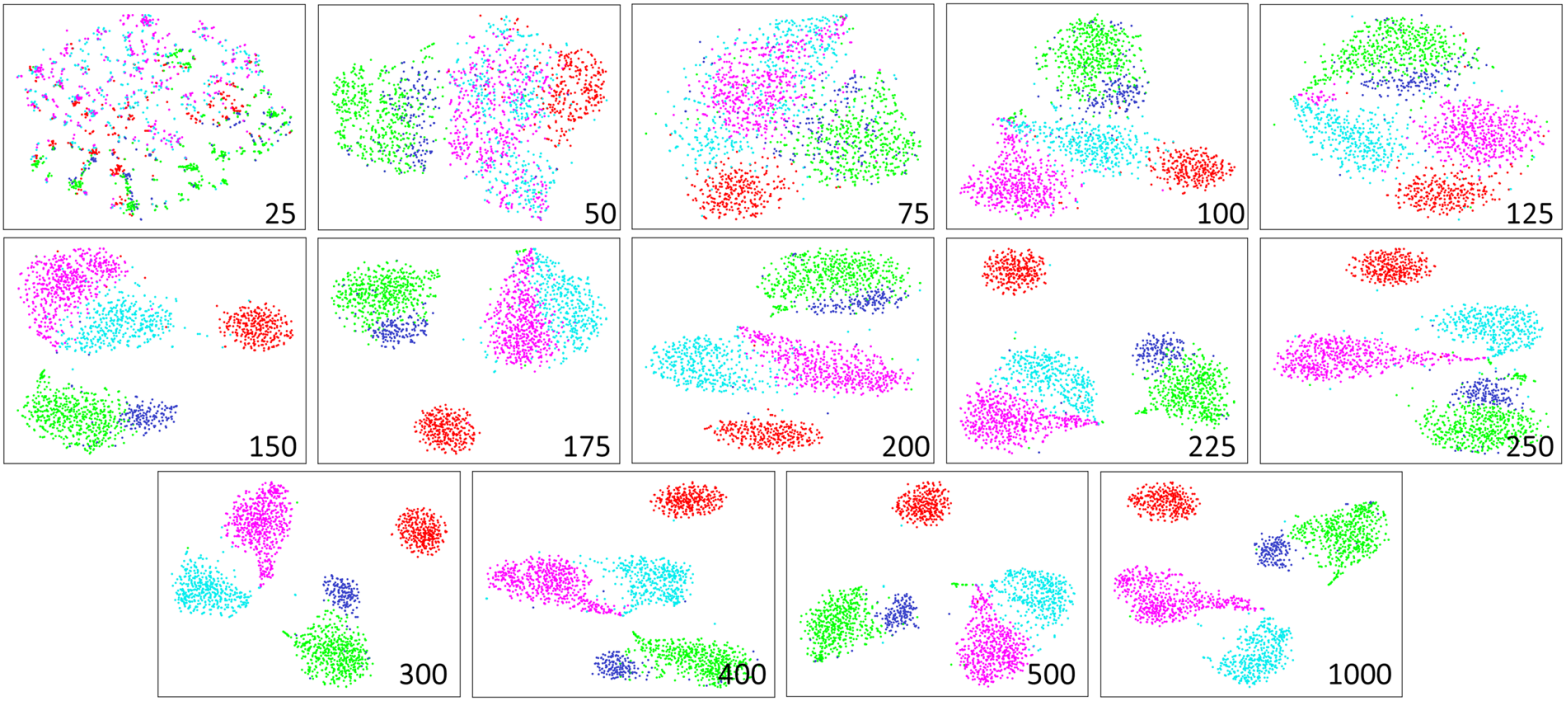
Background Tumor Classification Potential Revealed by Random Transcript Subsets of Sufficient Size. t-SNE embeddings of random subsets of the tumor gene expression matrix are presented at various sample sizes. Top row, left to right: subsets of size 25, 50, 75, 100, 125; middle row: subsets of size 150, 175, 200, 225, 250; bottom row: subsets of size 300, 400, 500, 1000. Sample colors are as follows: BLCA, light blue; GBM, dark blue; LGG, green; OV, red; THCA, magenta.

To test if the effect of classification potential was simply due to the number of transcripts in the GEM, random samplings of transcripts from the TCGA matrix were taken to determine the tumor classification potential of random GEMs of increasing size (Fig. 8). Random samplings of 200 transcripts or less either produced indistinct clusters or clusters of mixed tumor type identity. Larger random samples of transcripts, when t-SNE embedded and HDBSCAN clustered, were generally better able to segregate the five tumor types. We term this random classification potential the “background classification potential” as opposed to the more specific biomarker classification potential seen in early iterations of DQC based feature selection.

## Discussion

The motivation for this study was to identify better methods for segregating samples into biologically-relevant groups based upon quantitative dimensions (i.e. steady state RNA expression). We expected that these dimensions would confer biological classification potential which in this study was the separation of tumor types without prior knowledge of the sample origin. To achieve our goal, we tested two approaches, DQC and t-SNE, both of which grouped samples into clusters that made biological sense based on sample annotation enrichment.

The biological relevance of the tumor clusters was evidenced by enrichment of annotations relative to all tumors. The consensus clustering technique applied to one thousand t-SNE embeddings revealed nine tumor subpopulations (Figs 3 and 4). For example, we observed in cluster 3 that BLCA and OV tumors were similar enough to co-cluster and be enriched for late onset (> = 40 years) whereas cluster 4 was late onset but restricted to OV tumors. The unusual co-clustering of disparate tumor types in cluster 3 warrants further investigation. Further, we identified four clusters (0, 5, 6, 8) that were detected in younger patients (< = 40 years) and two additional clusters of tumors found in older patients (1, 2). We also observed an enrichment in clusters 0 and 5 for THCA and female attributes. It should be noted that 59/572 (10.3%) of samples labeled THCA were actually “Solid Tissue Normal” samples. These clusters make sense as thyroid cancer is 2.9 times more likely to occur in females^22^. Clusters 1, 6, 8 showed a male bias and clusters 6 and 8 are associated with brain cancer; this finding corroborates the male bias seen in brain tumor incidence^23^. None of the brain tumor samples were annotated as normal. With a deeper attribute dataset, we believe this approach would reveal higher quality sample groups and even more insight for the exploration of tumor biology.

While both t-SNE and DQC performed well in clustering the tumors, there were some interesting differences. DQC revealed interesting patterns that were not observed with t-SNE. For example, the DQC “brain arch” driven by the 48 core transcripts revealed an interesting substructure connecting LGG and GBM brain tumors (Fig. 2). When we clustered the samples across the DQC arch from LGG to GBM, we found the 48 core transcripts expression levels varied across the “brain arch” and exhibited a trend where epithelial, thyroid, and other genes were often up-regulated in samples at the bottom of the left-hand leg of the arch (LGG group 1) and often down-regulated in samples at the bottom of the right-hand leg (GBM group 7). There also appears to be immune response, extracellular matrix, and differentiation genes turned on at the right-hand leg of the arch (GBM group 7) and off at the bottom of the left-hand leg (LGG group 1). These data suggest that the core 48 biomarkers have excellent classification potential for all tumors. Furthermore, a subset of these genes function differently in GBM and LGG tumors, which have very different survival times of 14.6 months (GBM^24^) versus 7 years (LGG^25^).

This is not the first study to identify biomarkers and classify TCGA tumor types. Martinez *et al*. identified eight transcriptional superclusters using unsupervised hierarchical clustering of expression profiles between tumor sub-types across twelve TCGA tumor types^26^. In contrast to our approach, their study analyzed the top 1500 genes and used prior knowledge of tumor type in their analysis whereas our study input the full GEM and sorted tumors without using tumor type knowledge in the sorting process. Li *et al*. examined TCGA RNAseq data using a classification strategy where they classified 9096 tumor samples from 31 tumor types using GA/KNN as classification engine^27^. That classification study used prior knowledge of tumor type in the classification process whereas our study was blind to the sample labels. In Hoadley *et al*., a cluster-of-cluster (COCA) technique was used on RNAseq profiles (and other tumor molecular measurements) to determine a molecular taxonomy of 12 cancer types, discovering 11 molecular signature-based subtypes^9^. Their study showed that tissue of origin labels was not always indicative of the tumor’s molecular basis. In contrast to our study, they only used the 6000 most variable genes in their analysis. It is also typical to use many differentially expressed genes to characterize tumor type or subtype. Ceccarelli *et al*., for example, used 2275 differentially expressed genes to build “molecular profiles” of LGG and GBM tumors^21^ and Verhaak *et al*. identified 840 genes predictive of subtype in the case of GBM tumors alone^28^. We are aware that the 48 core transcripts used in this analysis are probably a subset of such a larger population of classifier genes.

Our results confirm there are two means of classifying samples: (1) using a small number of transcripts of high significance or (2) many transcripts of low relative significance. By examining the functions of genes in the first iteration of DQC-based transcript selection (Fig. 7; Supplemental Table 2), it was clear that the first 48 core transcripts show more relevance to the tumor phenotype and thus are more likely to be mechanistic in tumor progression. We also demonstrated the existence of a “background classification” effect where a random sample of – on the order of 200 transcripts – recapitulates the classification potential of the transcripts identified by DQC-based feature selection (Fig. 8) and supports the convention of using sets of hundreds or thousands of differentially expressed genes to characterize tumor types. It is possible, then, that relaxing the transcript significance threshold used in DQC-based feature selection may not identify additional transcripts of interest so much as reveal the high classification potential arising from the aggregate small effects of many transcripts.

While the “background classification” effect suggests that a sufficiently large set of random gene expression vectors has classification potential, it seems unlikely that all these genes would be involved in tumor specific biology. We assumed that through successive levels of DQC-based transcript selection we would remove differentially expressed genes and detect essentially random genes without collective function that merely contain the background classification potential. In fact, we did find that as we continued this process, the number of pathways we detected decreased after correcting for the number of transcripts found in the pathway (Fig. 6B). However, functional enrichment analysis, as defined by Reactome biochemical pathway enrichment in a gene group relative to all genes in the genome^29^, revealed a repeating pathway enrichment pattern for iterations 2, 3, 4, 6, and 7. The pathways that appear in these iterations appear to control protein synthesis and may be a signal for general cell growth and proliferation processes. It is interesting to note that the threshold used in the DQC-based feature selection process for the first seven levels did not decrease significantly. Rather, the significant drop in threshold was only seen in higher iterations. The drop-off in the number of observed reaction pathways appears to coincide with the drop-off of the threshold that had to be used in the selection process. A future experiment could determine if these repeating genes and pathways are present in normal tissue GEMs (e.g. GTEX datasets^2^) which would imply tissue specificity as opposed to a tumor type property.

In conclusion, we describe and contrast two very different sample clustering algorithms: DQC and t-SNE. We implemented dimensionality reduction to identify the important transcripts and all subsequent DQC analysis was done without further dimensionality reduction. t-SNE analysis involved further embedding into two spatial dimensions. Both techniques are effective at clustering samples to detect substructures in a GEM. The fact that DQC worked in the full 48-dimension space of core transcripts is likely the reason it reveals more subtle aspects of the data. Unexpectedly, we discovered the confounding effect many random transcripts can classify and sort samples. We also repeatedly showed that this ability is not merely identifying tissue of origin as opposed to tumor type. This is an early but intriguing concept that should be addressed if a researcher seeks cause-and-effect as opposed to tumor type-associated biomarkers. Finally, while we applied these techniques to tumor data, the same approach can be applied beyond genomics contexts, a fact that has been previously shown for DQC^19^.

## Methods

### Gene Expression Matrix (GEM) Preparation

RNAseq profiles, rsem processed at the transcript level, for five public tumor types were downloaded on April 1, 2016 from the TCGA Data portal at https://gdc-portal.nci.nih.gov. A total of 2,016 datasets were obtained comprised of the types as labeled by TCGA: BLCA (n = 427), GBM (n = 174), LGG (n = 534), OV (n = 309), and THCA (n = 572). Each expression profile was merged into a single gene expression matrix (GEM) with 73,599 transcripts labeled with *knowngene*5 UC-Santa Cruz genome database gene model identifiers. It is important to address potential batch effects – technical and biological variation between samples of the same group – in a high throughput genomics study^6^. The TCGA Batch Effects webserver (http://bioinformatics.mdanderson.org/tcgambatch/) was queried to gain insight on batch effects present in each cancer subtype. It was found that the Dispersion Separability Criterion (DSC) scores for the RNAseqv2 isoform data for each cancer subtype indicate a mild presence of batch effects in the data used in our study (p < 0.0005). The DSC scores for BLCA, GBM, LGG, OV, and THCA are 0.310, 0.000, 0.298, 0.089, and 0.252, indicating a low ratio of dispersion between vs. within batches for these cancer types. Lauss *et al*. found that performing a quantile normalization on colon cancer RNAseqv2 data from TCGA helped to reduce batch effects present in this data^6^. We performed a similar quantile normalization on the GEM used in our study. Furthermore, we did not detect any outliers using a Kolmogorov–Smirnov test as performed in^8^. The TCGA GEM was quantile normalized and randomly sorted to create a single tumor GEM for input into the DQC and t-SNE pipelines. A small fraction of the subtype labeled samples (83 out 2016–4.12%; 19 BLCA; 59 THCA; 5 GBM) included “Solid Tissue Normal” samples. These and other clinical annotations associated with each TCGA sample are included in Supplemental Table 3.

### DQC-Based Important Transcript Selection

As DQC evolution proceeds it becomes apparent that sample data points separate well in some dimensions and not in others (see sample DQC tumor evolution animation in Supplemental Video 2). This separation allows for a novel form of feature selection where we select a subset of the RNA transcripts that play the most important role in the evolution of the data.

To describe the DQC selection process it is convenient to consider the transpose of the original GEM, so that the rows are the tumor samples and the columns are the RNA transcript labels. The SVD-decomposition of this matrix rewrites the *m* × *n* GEM (*m* samples, *n* RNA transcripts) as Equation (3):

3 $$GE{M}_{ij}=\sum _{l}{U}_{il}{S}_{ll}{V}_{lj}^{t}$$

where *U* is an *n* × *n* matrix, *S* is an *n* × *n* diagonal real matrix with non-vanishing entries only along the diagonal arranged in decreasing value, and *V*^*t*^ is an *m* × *n* matrix.

The rows of *V*^*t*^ are unit vectors in the *m-*dimensional space of transcripts and define a new coordinate system best adapted to plotting the data. As the corresponding eigenvalue of *S* goes down, the variance of the data in that direction drops as well. In general, the rows of *V*^*t*^ are linear combinations of the original features that correspond to columns in *GEM*. It is common to dimensionally reduce the matrix *GEM* by choosing an integer *N* that is smaller than the number of rows in *GEM* and defining Equation (4)

4 $$GE{M}_{ij}^{N}=\sum _{l=1}^{N}{U}_{il}{S}_{ll}\,{V}_{lj}^{t},$$

where the sum over *l* goes from 1 to *N*. The square root of the sum of the squares of the left-out terms in *S*_*ll*_ provide an upper bound on the absolute error one makes in the full data matrix by using the dimensionally reduced matrix, instead of the original matrix. It is very convenient, when doing DQC evolution to replace the dimensionally reduced matrix $GE{M}_{ij}^{N}$by *U*^*N*^, an *n* × *N* matrix made up of the first *N* columns of *U*.

Note that while the dimensionally reduced matrix *U*^*N*^ has fewer columns than the original *GEM* it does not depend on fewer features, since each row of *V*^*t*^ depends upon many more than *N* features.

DQC based feature selection simply examines those rows of *V*^*t*^ that correspond to directions where the DQC evolved data clearly separates. For each of these vectors, we plot the absolute value of the eigenvector components - sorted in decreasing order. These plots tend to show a close group of larger values, followed by a gap, followed by smaller values. We select those features corresponding to large values of the components of the row of *V*^*t*^ in question. We combined features identified in this way to arrive at our set of *selected features*.

We should emphasize that this simple feature selection technique requires prior DQC evolution of the data to identify the useful directions of the SVD-decomposition. It should be noted that no theorem guarantees this approach will produce an exhaustive list of most important transcripts and that no information about tumor types was used at any stage of the selection process.

### t-SNE Analysis and Consensus Cluster Detection

The full or partial GEM was evaluated separately by a dimensionality reduction (embedding), clustering, consensus, and enrichment pipeline. Embedding was performed using t-Distributed Stochastic Neighbor Embedding (t-SNE^13^) using the Python implementation from https://github.com/DmitryUlyanov/Multicore-TSNE. For each t-SNE run, one thousand two-dimensional randomly initialized embeddings were created. Each embedding was clustered individually using Hierarchical Density-Based Spatial Clustering of Applications with Noise (HDBSCAN^30^). A consensus of nine clusters was determined over the whole set of clustered embeddings using the Cluster Ensembles method^31^. Label enrichment of these consensus clusters for patient attributes associated with the tumor samples was evaluated using a Chi-squared test (p < 0.001).

### Background Classification Potential

Repeated samples, varying in size, of random subsets of transcripts were extracted from the TCGA matrix and embedded using t-SNE. Twenty random sample subsets of size 25, 50, 75, 100, 125, 150, 175, and 200, ten samples of subsets of size 225, 250, 300, 400, and 500, and five subsets of size 100 were evaluated. These were treated with the same pipeline as above where the samples were embedded, clustered with HDBSCAN, consensus clusters were assigned by Cluster Ensembles, and then each of these consensus clusters was evaluated for tumor type enrichment. The percentage of clusters enriched for at least one tumor type was recorded.

### Functional Enrichment Analysis

Functional enrichment of the core and subsequent iterations of transcripts was performed using an in-house Perl script modeled after the online DAVID tool at https://david.ncifcrf.gov. Tested attributes include human transcripts mapped to terms from InterPro^32^, PFAM^33^, the Gene Ontology (GO)^34^, the Kyoto Encyclopedia of Genes and Genomes (KEGG)^35^, Reactome^36^ and MIM^37^. Terms that were present in a gene list more often than in the genomic background were considered enriched (FDR <0.01). The full enrichment list is shown in Supplemental Table 2.

## Electronic supplementary material

Supplemental Video 1

Supplemental Video 2

Supplemental Table 1

Supplemental Table 2

Supplemental Table 3
